# Canine *Trypanosoma cruzi* infection in the Bolivian Chaco

**DOI:** 10.1186/s13071-018-3247-0

**Published:** 2018-12-12

**Authors:** Simona Gabrielli, Michele Spinicci, Fabio Macchioni, David Rojo, Valentina Totino, Patricia Rojas, Mimmo Roselli, Herlan Gamboa, Gabriella Cancrini, Alessandro Bartoloni

**Affiliations:** 1 Dipartimento di Sanità Pubblica e Malattie Infettive, Università di Roma Sapienza, Rome, Italy; 2 Dipartimento di Medicina Sperimentale e Clinica, Università degli Studi di Firenze, Florence, Italy; 3 Dipartimento di Scienze Veterinarie, Università degli Studi di Pisa, Pisa, Italy; 4 Escuela de Salud del Chaco Tekove Katu, Gutierrez, Plurinational State of Bolivia; 5 Distrito de Salud Cordillera Santa Cruz, Camiri, Plurinational State of Bolivia; 6 Facultad Integral del Chaco, Universidad Autónoma Gabriel René Moreno, Camiri, Plurinational State of Bolivia

**Keywords:** *Trypanosoma cruzi*, Chagas disease, Dogs, Humans, Bolivia

## Abstract

A cross-sectional study on *Trypanosoma cruzi* was carried out in 2013 to evaluate the role of dogs as possible source of infection for humans in two rural communities of the highly endemic Bolivian Chaco (Bartolo, Chuquisaca Department, *n* = 57 dogs; and Ivamirapinta, Santa Cruz Department, *n* = 48 dogs). Giemsa-stained thick and thin smears, rapid immunochromatographic test (ICT) (Chagas Quick test, Cypress Diagnostic, Belgium) and polymerase chain reaction for *T. cruzi* on dried blood spots were performed. All smears proved negative by microscopic examination, whereas 23/103 (22%) were positive by ICT and 5/105 (5%) blood samples contained *T. cruzi* DNA, evidencing the potential role of dogs in the domestic transmission of the parasite.

## Letter to the Editor

Chagas disease (CD) (or American trypanosomiasis), caused by the protozoon *Trypanosoma cruzi,* remains a major public health threat in Latin America, affecting an estimated 6–7 million individuals, of whom 30–40% either have or will develop cardiomyopathy, digestive megasyndromes, or both [1]. Vector-mediated route is the main pathway of CD transmission in rural areas, where Triatominae (Hemiptera: Reduviidae) typically live into cracks and holes of walls, ceilings and floors of mud and straw houses, feeding on both humans and domesticated animals [2]. Over 100 different mammal species are competent reservoirs of *T. cruzi*, implicated in the sylvatic transmission cycles in nature [3]. Canines play an important role as reservoir hosts in the peridomestic circulation and act as a bridge between sylvatic and domestic transmission cycles [4]. Humans can also acquire the infection *via* congenital, transfusion/transplantation and/or oral routes [5, 6].

Several endemic countries of South America achieved a substantial reduction in CD incidence thanks to control interventions, mainly focused on domestic vector elimination, and prevention of transfusion transmission infections, whereas the region of Gran Chaco (southern Bolivia, northern Argentina and western Paraguay) remains at high risk for *Triatoma* transmission [7–10]. As for the Bolivian Chaco, cross-sectional surveys carried out in 2011–2012 in the Eiti health sector (Department of Santa Cruz) documented an extremely high seroreactivity to *T. cruzi* in humans, up to 20% among 2–15 years-old children, 73% among participants aged 15–30 years, and 97% among participants older than 30 years [11]. In that study area, more than 90% of the housholds owned a domestic dog, but no data on *T. cruzi* canine infection were available, leading the authors to warrant further assessments about the possible role of dogs as infection reservoirs [11]. In 2013, another cross-sectional survey, that took place in two rural communities (Ivamirapinta, Department of Santa Cruz and Bartolo, Department of Chuquisaca), confirmed the high risk of CD transmission in this area, both *via* vectorial and vertical route (seroprevalence for *T.cruzi* ranged between 24–29% among < 20 year-old participants and between 74–79% among women of reproductive age) (Spinicci M et al., unpublished data).

During the last-mentioned survey, a serological and molecular screening for *T. cruzi* in domestic dogs was also performed, in order to evaluate their role as possible source of transmission of *T. cruzi* to humans in the surveyed communities. The communities of Bartolo and Ivamirapinta are located in the Municipality of Monteagudo, Hernando Siles Province, Chuquisaca Department (16°30'S, 59°88'W) and in the Municipality of Gutierrez, Cordillera Province, Santa Cruz Department (19°45'S, 63°30'W), respectively. In both communities, households are typical rural dwellings, predominantly constructed of mud and sticks or adobe, with packed dirt floors, and straw or corrugated metal roofs; local economy is based on subsistence farming and animal husbandry. A total of 105 dogs were consecutively enrolled, representing ≈50% and ≈25% of the canine populations of Bartolo and Ivamirapinta, respectively. Blood samples were collected by venepuncture, according to the international guiding principles for biomedical research involving animals, issued by the Council for International Organizations of Medical Sciences [12]. Local inhabitants helped the investigators to manipulate the animals during sampling, in order to minimize the risk of incidents.

Blood samples were processed and Giemsa-stained thick and thin smears were prepared, a rapid immunochromatographic test (ICT) (Chagas Quick test, Cypress Diagnostic, Langdorp, Belgium) was performed, and impregnated filter papers using 300–500 μl of blood were prepared. DNA was extracted from filter papers using the Dried Blood Spot Genomic DNA Isolation Kit (Norgen Biotek Corp, Thorold, Canada) according to the manufacturer’s instructions and subsequently submitted to a TaqMan RT-PCR assay to amplify a region of the *18S* rRNA gene of *Trypanosoma cruzi* (Genesig PrimerDesign, Camberley, England).

Data were entered into Microsoft Excel 2010 software (Microsoft, Redmond, WA, USA). Statistical analysis of the data was performed with STATA 11.0 (StataCorp, College Statio, TX, USA). Frequencies and percentages with 95% confidence intervals (CI) for categorical variables, medians and interquartile ranges (IQR) for continuous variables were calculated. Person chi-square test, or Fisher’s exact test when appropriate, were performed to investigate statistical associations. *P*-values < 0.05 were considered significant.

Blood samples were collected from 57 dogs living in Bartolo (28 females out of 53 dogs with sex data available, 53%) and 48 from Ivamirapinta (23/48, 48% females). The median age of dogs was 5.7 years (range 6 months to 11 years). All blood thick and thin smears proved negative by microscopic observation, whereas 5 samples were positive for *T. cruzi* by PCR. In detail, 3/57 (5.3%, 95% CI: 0.5–11.1%) and 2/48 (4.2%, 95% CI: 1.5–9.8%) were positive in Bartolo and Ivamirapinta, respectively (Fisher’s exact test: *P* = 0.582). Seroprevalence for *T. cruzi*, obtained by ICT, was higher in Bartolo (15/55, 27.3%, 95% CI: 15.5–39.0%) than in Ivamirapinta (8/48, 16.7%, 95% CI: 6.1–27.2), the difference being non-significant (Chi-square test: *χ*^2^ = 1.66, *df* = 1, *P* = 0.197).

Our study, carried out in two rural communities of the Bolivian Chaco, characterized by a very high endemicity in humans (*T. cruzi* seroprevalence in 2013: 60% in Ivamirapinta and 66% in Bartolo; Spinicci M et al., unpublished data) confirmed the potential role of dogs in the domestic transmission of *T. cruzi*. Our results are in line with previously reported canine seroprevalence data in the Americas, which typically ranged between 10–30% [4]. In Bolivia, a similar prevalence was reported in the department of Santa Cruz and Cochabamba, by using both xenodiagnosis (XD) (23.4%) and serology testing (23.5%), whereas lower values were observed from other XD-based studies (6–8%) [13–16]. As for the Argentinian Chaco, a similar range of prevalence was reported in recent years (11.3–27.6%) [17, 18]. The inadequate housing structures and the poor living conditions, in addition to the lack of systematic vector control interventions, fostered a steady *T. cruzi* circulation within the Bolivian Chaco. Dogs, as well as cats, commensal rodents and domesticated guinea pigs, play key roles as amplifying hosts and sources of *T. cruzi* in many (peri) domestic transmission cycles covering a broad diversity of ecotopes and triatomine species. Dogs comply with the desirable attributes of natural sentinels and sometimes are a point of entry of sylvatic parasite strains, as they usually are neither supervised nor their movements restrained across several rural areas; they have free access to human sleeping quarters and rest in proximity to humans. In addition, general conditions of dogs, as malnutrition, could enhance the parasite reproduction in the bloodstream. Therefore, these animals represent a relevant domestic reservoir of *T. cruzi* as high prevalence rates of dog infection were detected in several endemic areas, sometimes reaching or exceeding the local human infection rates [4].

This study has several limitations including the cross-sectional design, the limited size of dog samples, the lack of data concerning entomological collections and *T. cruzi* discrete typing units (DTU) distribution. Moreover, information about the host-feeding patterns of domestic bugs, the infectiousness of dogs to bugs and the attractiveness to bugs of other animals living in the surveyed areas are missing. Further studies are warranted to better explore these issues.

In conclusion, *T. cruzi* transmission is still a major health concern in the Bolivian Chaco and the presence of multiple animal reservoirs, including canine population, was confirmed as a further challenge for disease control and prevention strategies.

## Abbrevations

CD: Chagas disease
CI: confidence interval
ICT: immunochromatographic test
PCR: polymerase chain reaction
XD: xenodiagnosis
